# Does the time between CT scan and chemotherapy increase the risk of acute adverse reactions to iodinated contrast media in cancer patients?

**DOI:** 10.1186/1471-2407-14-792

**Published:** 2014-10-31

**Authors:** Alberto Farolfi, Elisa Carretta, Corradina Della Luna, Angela Ragazzini, Nicola Gentili, Carla Casadei, Domenico Barone, Martina Minguzzi, Dino Amadori, Oriana Nanni, Giampaolo Gavelli

**Affiliations:** Department of Medical Oncology, Istituto Scientifico Romagnolo per lo Studio e la Cura dei Tumori (IRST) IRCCS, via Piero Maroncelli 40, Meldola, 47014 Italy; Unit of Biostatistics and Clinical Trials, IRST IRCCS, via Piero Maroncelli 40, Meldola, 47014 Italy; Oncology Pharmacy, IRST IRCCS, via Piero Maroncelli 40, Meldola, 47014 Italy; IT Unit, IRST IRCCS, via Piero Maroncelli 40, Meldola, 47014 Italy; Anesthesiology Unit, IRST IRCCS, via Piero Maroncelli 40, Meldola, 47014 Italy; Radiology Unit, IRST IRCCS, via Piero Maroncelli 40, Meldola, 47014 Italy

**Keywords:** Iodinated contrast media, Time to adverse reaction, Hypersensitivity, Contrast-enhanced CT, Cancer patients and chemotherapy

## Abstract

**Background:**

Cancer patients undergo routine computed-tomography (CT) scans and, therefore, iodinated contrast media (ICM) administration. It is not known whether a time-dependent correlation exists between chemotherapy administration, contrast enhanced CT and onset of acute ICM-related adverse reactions (ARs).

**Methods:**

All consecutive contrast-enhanced CTs performed from 1 January 2010 to 31 December 2012 within 30 days of the last chemotherapy administration were retrospectively reviewed. Episodes of acute ICM-related ARs were reported to the pharmacovigilance officer. We analyzed time to CT evaluation calculated as the time elapsed from the date of the CT performed to the date of the last chemotherapy administration. Patients were classified into 4 groups based on the antineoplastic treatment: platinum-based, taxane-based, platinum plus taxane and other group.

**Results:**

Out of 10,472 contrast-enhanced CTs performed, 3,945 carried out on 1,878 patients were considered for the study. Forty acute ICM-related ARs (1.01%; 95% CI, 0.70-1.33) were reported. No differences were seen among immediate (within 10 days of the last chemotherapy administration), early (11–20 days) and delayed (21–30 days) CTs. Median time to CT in patients who experienced an acute ICM-related AR by treatment group was not statistically different: 20 days (range 6–30), 17 days (range 5–22), 13 days (range 8–17), 13 days (range (2–29) for the platinum, taxane, platinum plus taxane and other group, respectively (*P* =0.251).

**Conclusions:**

Our results did not reveal any correlation between time to CT and risk of acute ICM-related ARs in cancer patients.

## Background

Cancer mortality in Western countries has steadily declined in recent years [1]. However, differences in survival have been identified between European countries, possibly due to variations in socioeconomic circumstances, lifestyle and general health between the different populations. The likely explanations include differences in stage of diagnosis and accessibility to good care, different diagnostic intensity and screening approaches in the need for more prolonged patient follow-up [1]. In this context, the need to assess the extent of the disease (staging), the response to treatment and the follow-up of cancer patients has resulted in the need for more radiological examinations, particularly contrast-enhanced computed tomography (CT) scans. Consequently, cancer patients may be at higher risk of suffering acute allergic-like adverse reactions (ARs) to iodinated contrast media (ICM) [2].

In the general population, the incidence of likely acute ICM-related ARs with low-osmolar contrast media is 0.2%-0.7% and approximately 0.04% for severe reactions [3–6] In cancer patients, acute ARs may occur in 0.93% of examinations, with 0.1% being severe [2].

Although it is clear that certain patients are at increased risk of experiencing ICM-related ARs, contrast reactions remain sporadic and unpredictable. It should also be considered that all main systemic agents used in cancer treatment today are associated with possible hypersensitivity reactions, although there are differences among agents in terms of the onset time of the symptoms [7, 8]. Since both ICM and taxanes may induce reactions at first administration [9], it may be assumed that both drugs act via similar immune-mediated mechanisms. In order to determine the risk/benefit ratio on the time of evaluation of the disease and to evaluate if the risk may be time-dependent, we analysed the time elapsing between the contrast-enhanced CT scan, the most recent chemotherapy administration and the onset time of the ICM-related ARs in a consecutive cohort of cancer patients.

## Methods

### Patient population

All consecutive contrast-enhanced CT scans performed at IRST IRCCS (Meldola, Italy) from 1 January 2010 to 31 December 2012 were retrospectively reviewed. Only one CT examination per person per day was evaluated. The hospital, which is an institute for cancer research and treatment, deals only with adult cancer patients. The analysis included patients enrolled on an observational trial which has previously been reported [2]. Briefly, for each patient, we collected: date of birth, gender, primary tumor, date of diagnosis, anti-neoplastic therapy, type and setting of anti-neoplastic therapy (adjuvant/neoadjuvant or advanced), treatment start and end date, date of last cycle and date of contrast-enhanced CT scan. All the therapies administered in the 24 months before the date of the CT scan were analysed. The study protocol was reviewed and approved by the Medical Scientific Committee and the local Ethics Committee of IRST IRCCS. For this study, we analysed all contrast-enhanced CTs carried out within 30 days from the most recent chemotherapy administration.

### CT scanning and ICM administration

All CTs, most predominantly of the thorax and abdomen, were performed using a 256-slice CT scanner (Brilliance iCT, Philips Healthcare S.p.A., Milan, Italy). Written informed consent for the use of ICM was obtained from all patients. Patients fasted for at least 6 hours before the examination and were encouraged to drink abundantly for 24 hours before, unless contraindicated.

In the preparation room, the anaesthesiologist checked the signed informed consent form and the presence of any contraindications to the ICM injection. Two main low-osmolar ICMs were used at our institution: during the study period, the consumption of ICM was 80% for iomeprol (Iomeron®, 300–400 mgI/ml) and 20% for iobitridol (Xenetix®, 300–400 mgI/ml). All ICM injections were monitored by anaesthesiologists and all radiologists were certified in basic life support.

In relation to acute allergic-like ARs, in the case of severe ICM-related ARs, patients are advised to undergo another form of imaging, whenever possible. Patients with any history of severe bronchospasm, severe laryngeal oedema, angioedema, unresponsiveness, seizure activity, cardiac arrhythmias or cardiopulmonary arrest following the administration of at least one drug, or a history of allergies to more than one drug, or reported mild or moderate ICM-related ARs are advised to undergo premedication, as previously stated [2].

### Outcome measures

The attending CT radiologist and anaesthesiologist recorded any ARs observed by them or described by patients during the ICM injection or for one hour afterwards, which were then subsequently recorded by the hospital pharmacist (CDL), responsible for pharmacovigilance. CTs performed after an AR were excluded from the analysis.

Time of treatment was calculated as the time between the first day of the first cycle of chemotherapy and the last day of administration. Time to CT evaluation was calculated as the time elapsing from the date of the CT and the date of most recent chemotherapy administration. Patients were classified into four groups based upon the anti-neoplastic treatment received: “platinum” group in the case of a platinum-based regimen (oxaliplatin, carboplatin or cisplatin), “taxane” group in the case of a taxane-based regimen (either docetaxel or paclitaxel), “platinum plus taxane” group if the chemotherapy included both drugs and the “other” group (any chemotherapy without platinum or taxane). Patients were excluded if they had not been treated with an anti-neoplastic treatment during the 24 months preceding the CT scan.

### Statistical analysis

Categorical variables are expressed as frequencies and percentages, and continuous variables are reported as means (standard deviation, SD) or median (range). The incidence of acute ICM-related ARs and CT variables (time of treatment, time to CT, patient number of CT) were compared using the Kruskall-Wallis test or Chi-Square and Fisher’s exact tests as appropriate. Univariate logistic regression was performed to calculate the risk of ICM-related ARs for time to CT evaluation and treatment groups. Statistical significance was set at *P* <0.05, and all reported p values are two-tailed. All data was analysed using SAS software, version 9.3 (SAS Institute, Cary, NC).

## Results

### Study population

During the study time period, 10,472 contrast-enhanced CT scans were performed on 3,804 patients. For this study, we analysed all contrast-enhanced CT scans carried out within 30 days from the most recent chemotherapy administration. From 10,472 contrast-enhanced CT scans, 3,945 examinations performed on 1,878 patients were considered eligible. The median age of the population was 63 years (range 18–91 years), with 51.2% being male. The mean number of contrast-enhanced CT scans in the study period was 2.13 (SD 1.59), with a median time to CT of 13 days (range 1–30).

**Figure 1:**
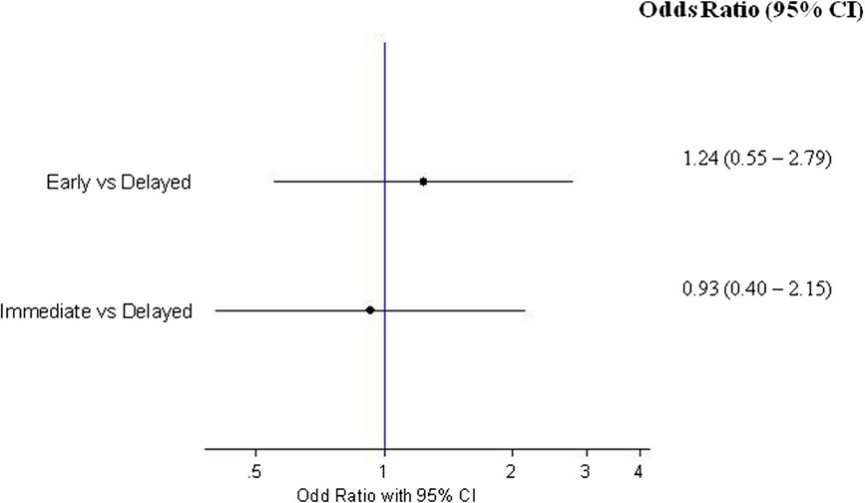
**Forrest plot of the risk of ICM-related ARs by time to CT evaluation.** Immediate (within 10 days of the last chemotherapy administration), early (from 11 to 20 days) and delayed (from 21 to 30 days) contrast-enhanced CT.

Out of 3,945 contrast-enhanced CT scans, 40 acute ICM-related ARs (1.01%; 95% CI, 0.70-1.33) were reported during the study time period. We then compared the risk of ICM-related ARs between immediate (within 10 days from the most recent chemotherapy administration), early (from 11 to 20 days) and delayed (from 21 to 30 days) contrast-enhanced CT. No differences were seen among these time periods, as shown in Figure 1. One hundred and eighty five patients underwent chemotherapy and contrast-enhanced CT scans on the same day, four of whom (2.16%; 95% CI, 0.07- 4.26) developed an acute ICM-related AR.

### Treatment groups

**Figure 2:**
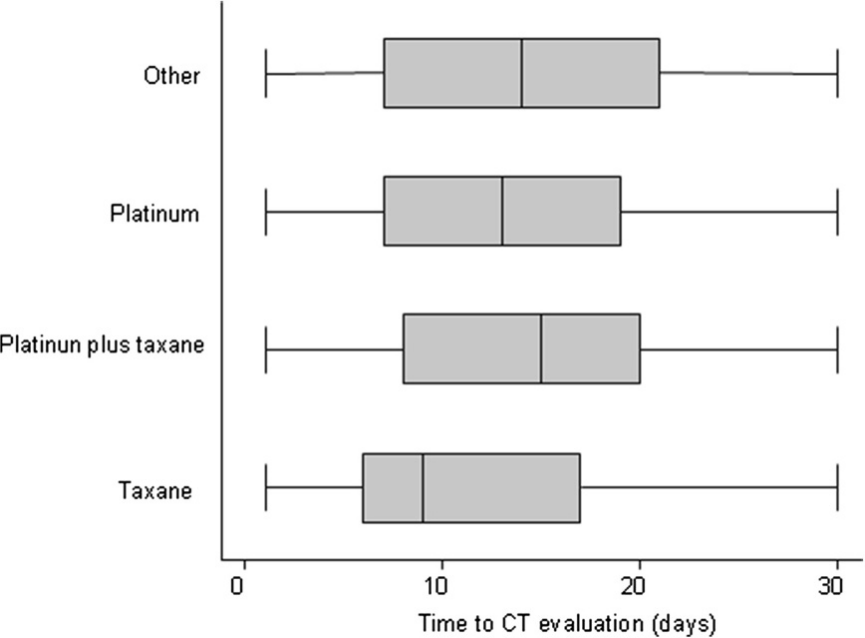
**Box-plot graphs for time to CT evaluation according to treatment groups.** Box-plots show the 25th and 75th percentile range (box) and median values (transverse lines in the box).

A descriptive analysis of characteristics of the study sample by treatment group is shown in Table 1. The median time of treatment was similar among the groups, a part of platinum plus taxane group that showed the shortest median time of treatment (50 days, range 1–258) (*P* <0.001). A difference between the groups in terms of median time to CT was seen (*P* <0.001), with the taxane group which had the shortest time (median 9 days, range 1–30), whereas the platinum plus taxane group had the longest (median 15 days, range 1–30), as shown in Figure 2.

**Table 1:** **Characteristics of study sample by treatment group**

	Platinum	Taxane	Platinum plus Taxane	Other	***P***
Contrast-enhanced CTs, n	1249	385	182	2129	
Median duration of treatment, d [range]	70 [1–502]	77 [1–483]	50 [1–258]	73 [1–707]	< 0.0001
Mean no. of CTs per patient (SD)	1.72 (1.07)	2.18 (1.59)	1.55 (0.78)	2.42 (1.82)	< 0.0001
Median patient age at CT, y [range]	65 [18–87]	64 [23–86]	61 [23–80]	66 [21–91]	< 0.0001
Primary tumor					
Gastrointestinal	618 (49.5%)	12 (3.1%)	27 (14.8%)	541 (25.4%)	< 0.0001
Urogynecological	160 (12.8%)	129 (33.5%)	115 (63.2%)	266 (12.5%)	
Breast	47 (3.8%)	176 (45.7%)	8 (4.4%)	523 (24.6%)	
Lung	305 (24.4%)	50 (13%)	10 (5.5%)	271 (12.7%)	
Hematological	6 (0.5%)	1 (0.3%)	2 (1.1%)	207 (9.7%)	
Melanoma	44 (3.5%)	-	5 (2.8%)	128 (6%)	
Other sites	69 (5.5%)	17 (4.4%)	15 (8.2%)	193 (9.1%)	
ICM-related ARs, n (%)	9 (0.7)	5 (1.3)	4 (2.2)	22 (1.0)*	0.221

*Abbreviations:* Other, any chemotherapy without platinum or taxane; *n* number, *d* days, *y* years, *CT* computed tomography, *SD* standard deviation, *ICM* iodinated contrast media, *AR* adverse reaction.

*4 ARs with vinca alkaloid-based therapy; 3 with anthracycline-based therapy; 3 with fluorouracil-based therapy; 3 with tyrosine kinase inhibitors; 2 with IL2; 2 with gemcitabine; 1 with irinotecan; 1 with fotemustine; 1 with bevacizumab; 1 with interferon; 1 with ipilimumab.

**Figure 3:**
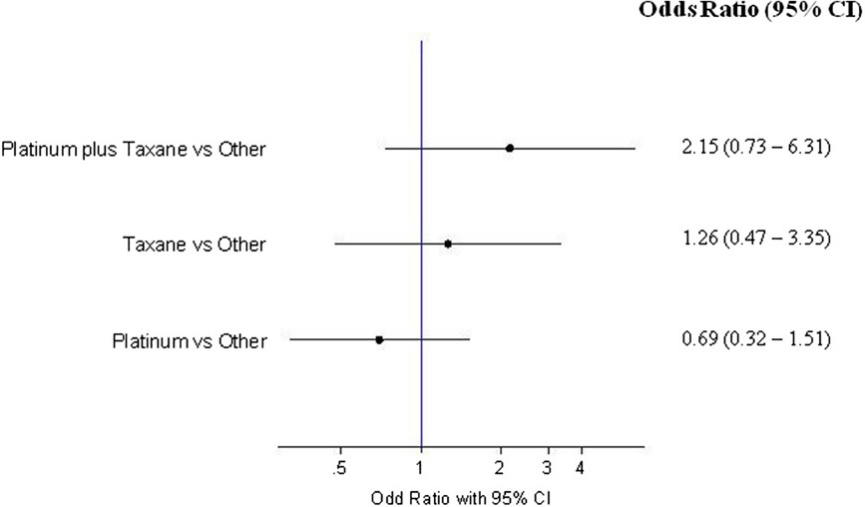
**Risk of ICM-related ARs by treatment group.**

The highest incidence of acute ICM-related ARs was seen in the platinum plus taxane group (2.2%; 95% CI, 0.07-4.33), followed by the taxane group (1.3%; 95% CI, 0.17-2.43). The risk of this event did not differ among the treatment groups considering the other group as a comparator (Figure 3). The mean number of CTs in patients with an acute ICM-related AR by treatment group was: 1.67 (SD 0.71), 1.4 (SD 0.55), 1.25 (SD 0.5) and 2.36 (SD 2.19), for platinum, taxane, platinum plus taxane and the other group, respectively (*P* =0.469). The median time to CT in patients who experienced an acute ICM-related AR by treatment group was not statistically different: 20 days (range 6–30), 17 days (range 5–22), 13 days (range 8–17), 13 days (range 2–29) for platinum, taxane, platinum plus taxane and the other group, respectively (*P* =0.251).

## Discussion

Millions of radiological examinations assisted by intravascular ICM are conducted each year. Although adverse side effects are infrequent [3–6], detailed knowledge of the variety of side effects, their likelihood of occurrence in relation to pre-existing conditions and information on their best form of treatment is required to ensure optimal patient care. Allergic-like reactions to ICM manifest in a similar way to true allergic reactions seen with other drugs and allergens; however, because an antigen-antibody response cannot always be identified, acute ICM-related ARs are classified as “anaphylactoid”, “allergic-like” or “idiosyncratic” [10–12].

It is worth noting that about a third of patients who have an ICM-related AR have not been exposed to ICM previously [12], suggesting that the etiological mechanisms of anaphylactoid contrast reactions seems to be related to the direct activation of mast cells [13]. Similarly, taxane-induced hypersensitivity can occur without prior sensitisation in more than half of cases [9], suggesting that both drugs may act via similar immune-mediated mechanisms.

We previously demonstrated that there was an increased risk of the onset of an acute ICM-related AR in patients previously treated with a taxane-based chemotherapy [2]. It could be speculated that taxanes may predispose to a subsequent allergic reaction to ICM because of the similar pathomechanism involved. In this context, the only substantial and validated risk factor for a recurrent AR was demonstrated to be a prior allergic-like reaction to ICM [3, 5, 14].

In this study we aimed to identify if a time-dependent correlation exists between chemotherapy administration, contrast-enhanced CT and the onset of acute ICM-related ARs. However, we failed to demonstrate that a shorter time interval leads to a higher risk of the event both in the global population and in considering the different treatment groups. However, the frequency of acute ICM-related ARs was high, especially in the platinum plus taxane and taxane groups, albeit not statistically significant, probably because this analysis excludes contrast-enhanced CT scans performed after 30 days from the most recent chemotherapy administration, with a consequently low number of events.

The absence of time-dependency in the risk of acute ICM-related ARs is reasonable from an immunological point of view. In fact, the iodine atoms on the benzene rings common amongst the various ICMs attach to the Fc (constant) portion of the IgE molecules and not the Fab (variable) portion, where most antigens attach. Mast cell release occurs when the IgE receptors aggregate when there is low ICM concentration attachment with or without the presence of antigen attachment [15]. Moreover, it is assumed that the activation of the complement and contact systems may be a further pathomechanism involved [16]. This is in line with the observation of acute ICM-related ARs that may also occur at first ICM administration. However, ICMs are rapidly eliminated, unchanged, through urinary excretion [17]. Hence, it may be assumed that the risk is somehow higher when possible allergens are administered in this period.

We acknowledge that our study has some limitations. In common with all retrospective studies, it is possible that we may have missed some data. However, the policy of our institution requires any AR to be reported to the pharmacovigilance manager. Moreover, we analysed a consecutive cohort of cancer patients, in order to mitigate the retrospective nature of the study. The number of side effects observed was limited, which may raise some concern over the strength of the observations. However, we consider our study as hypothesis generating, since no data exists in such a population and it definitely requires confirmation by way of other studies. Finally, we used a system for classifying patients into treatment groups that has intrinsic biases. Despite these limitations, we believe that some type of classification is necessary in order to evaluate the relationship between the anti-neoplastic treatment and ICM-related ARs.

## Conclusions

Around 1% of oncology patients receiving treatment may develop an acute ICM-related AR. However, we failed to demonstrate a time dependency between the time to CT and the risk of acute ICM-related ARs. This is in line with the proposed mechanism involved which is supposedly directly caused by histamine release from basophils and mast cells. Further research is needed to confirm our data and to understand the pathophysiologic action on the basis of this event.
